# Novel deep learning hybrid models (CNN-GRU and DLDL-RF) for the susceptibility classification of dust sources in the Middle East: a global source

**DOI:** 10.1038/s41598-022-24036-5

**Published:** 2022-11-11

**Authors:** Hamid Gholami, Aliakbar Mohammadifar

**Affiliations:** Department of Natural Resources Engineering, University of Hormozgan, Bandar-Abbas, , Hormozgan Iran

**Keywords:** Environmental sciences, Natural hazards

## Abstract

Dust storms have many negative consequences, and affect all kinds of ecosystems, as well as climate and weather conditions. Therefore, classification of dust storm sources into different susceptibility categories can help us mitigate its negative effects. This study aimed to classify the susceptibility of dust sources in the Middle East (ME) by developing two novel deep learning (DL) hybrid models based on the convolutional neural network–gated recurrent unit (CNN-GRU) model, and the dense layer deep learning–random forest (DLDL-RF) model. The Dragonfly algorithm (DA) was used to identify the critical features controlling dust sources. Game theory was used for the interpretability of the DL model’s output. Predictive DL models were constructed by dividing datasets randomly into train (70%) and test (30%) groups, six statistical indicators being then applied to assess the DL hybrid model performance for both datasets (train and test). Among 13 potential features (or variables) controlling dust sources, seven variables were selected as important and six as non-important by DA, respectively. Based on the DLDL-RF hybrid model – a model with higher accuracy in comparison with CNN-GRU–23.1, 22.8, and 22.2% of the study area were classified as being of very low, low and moderate susceptibility, whereas 20.2 and 11.7% of the area were classified as representing high and very high susceptibility classes, respectively. Among seven important features selected by DA, clay content, silt content, and precipitation were identified as the three most important by game theory through permutation values. Overall, DL hybrid models were found to be efficient methods for prediction purposes on large spatial scales with no or incomplete datasets from ground-based measurements.

## Introduction

Dust storms have significant effects on the terrestrial and marine environments, climate, biogeochemical cycle, socio-economic aspects of human societies, and their health^1–5^. Such storms are a serious threat in different countries and regions of Asia (e.g., west Asia^6,7^, China^8^, Iran^9^, and central Asia^10^), and especially in the ME^11^. Therefore, a susceptibility classification of dust sources is deemed necessary for the mitigationof the on-site (e.g., soil degradation, abrasion damage, and other damages consisting of loss of seeds, burial of plants, and etc.) and off-site adverse effects of dust storms.

Dust sources can be controlled by climatic factors, ground and land characteristics (e.g., vegetation cover, topographic variables, land surface roughness, and soil properties)^12,13^. Therefore, among variables suggested to represent dust source controls, identifying the influential or important variables is the key to accurate spatial modeling. Unfortunately, there is a general dearth of literature on the use of feature selection algorithms in the aeolian realm^14,15^. One study applied the leave-one-feature-out (LOFO) algorithm for identifying the influential variables controlling dust sources in central Asia^10^. In another work^16^, three variables comprising precipitation, digital elevation model, and soil organic carbon were identified as the key variables controlling dust emissions in southeastern Iran by the Boruta algorithm. Soil, geomorphology, and slope were identified as the three critical variables controlling dust sources in the Sistan watershed (Iran-Afghanistan borders) by two statistical-based predictive models, including frequency ratios (FR) and weights of evidence (WOE)^15^. In the present study, we applied the Dragonfly optimization (FDO) algorithm (a technique less applied to feature selection) to identify influential variables controlling dust provenance in the study area.

Deep learning (DL) models—a new generation of the machine learning (ML) models—are sophisticated tools and promising techniques for prediction purposes at different spatial and temporal scales^17,18^. Among the DL models, the most typical include SAE, DBN, CNN, and RNN models^19^. DL models are more efficient for prediction purposes and spatial modeling because they overcome many of the restrictions placed on the modeling process by traditional models^14,17^.

The application of the DL models (e.g., CNN, LSTM, GRU, etc.) has been reported frequently in different fields such as computer science, detecting anomaly, human interaction recognition^20–24^, etc., but its application in aeolian geomorphology is very new. For example, the successful application of two DL models consisting of RNN and RBM reported for spatial modeling of land susceptibility to dust emissions in the Kerman province, southeastern Iran^16^. In another work^10^, two DL models (e.g., gcForest and Bi-LSTM) and a copula-gcForest hybrid model were applied to mapping dust sources in central Asia.

To the best of our knowledge, application of individual CNNs or DL hybrid architectures for the classification of dust sources has not been reported in the literature before. Given the above context, our research applies, for the first time, the Dragonfly algorithm (DA) for discriminating the important features controlling dust source from the non-important features. In a second step, two DL hybrid models were developed based on the convolutional neural network and gated recurrent unit (CNN-GRU model) models, and a dense layer deep learning and random forest (DLDL-RF model) model to classify the spatial sources of dust in the ME. In a third step, six statistical indicators and game theory were used to assess the performance of the DL hybrid predictive model and its interpretability, respectively.

## Materials and methods

### Case study

**Figure 1:**
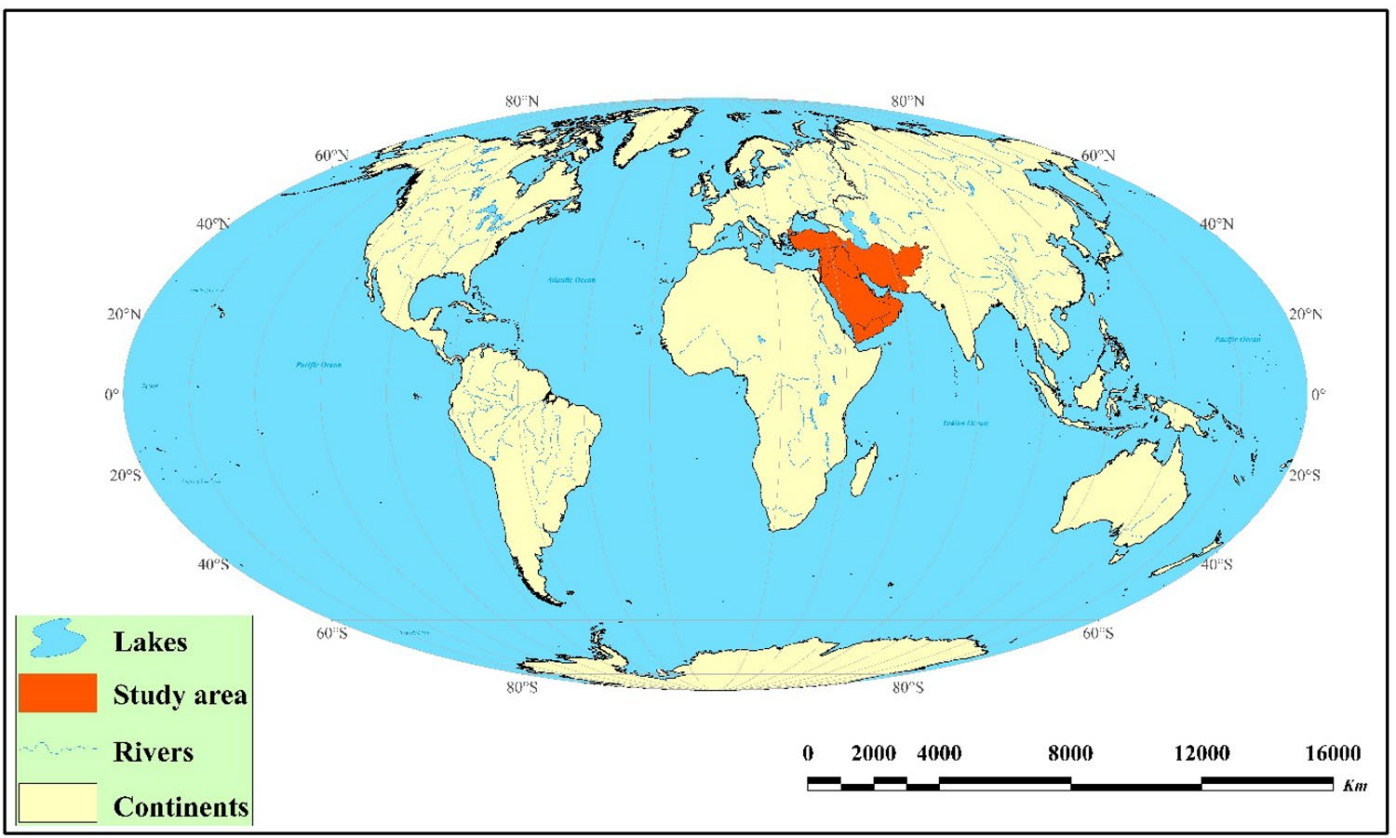
Location of the ME on the globe. This map was generated in ArcGIS 10.4.1 (https://www.esri.com/en-us/about/about-esri/overview).

The Middle East includes Iran, Afghanistan, Turkey, Iraq, Syria, Kuwait, Bahrain, Saudi Arabia, Jordan, Oman, Yemen, United Arab Emirates, and Qatar. With an area of 6.69 × 10^6^ km^2^, the ME is one of the critical sources of dust storms in the global system (Fig. 1). Dust storms are a serious threat especially in the drylands of the ME^25^. The key dust-bearing winds in the study area involve the Shamal (north wind), the Sad-ou-bist bad (wind of 120 days), the Belat, the Simoom (poison wind), the Khamsin (fifty), and the Shlour. They affect the Persian Gulf, southeastern Iran (especially the Sistan plain), southeastern Arabia, Kuwait, Egypt, Syria, and the Lebanon, respectively^26^. The elevation in the study area ranges between −418 and 6774 m. The mean annual precipitation ranges from 4 to 2101 mm.

### Mapping factors controlling dust sources and its inventory

Almost none the ME countries have complete ground-based observational datasets. Remote sensing have high potential to monitor dust events^7^. In the present study we mapped 13 factors controlling dust sources, these comprising land-surface aspect, clay content, silt content, sand content, DEM, lithology, land use, organic carbon content, precipitation, saturated hydraulic conductivity, available water content, soil bulk density, and wind speed. All soil properties were extracted from soil grids, an open source global soil assessment system (https://soilgrids.org). A DEM with a resolution of 90 m was downloaded from the earth explorer. The long-term climatic variables were downloaded from the Worldclime website (www.worldclimate.org) and then mapped in ArcGIS. Land use was downloaded from the website https://maps.elie.ucl.ac.be. Overall, all input layers used for the modeling process were converted to a spatial resolution of 50 × 50 m. The spatial maps for potential variables controlling dust sources are presented in Supplementary Figs. (S1–S13).

**Figure 2:**
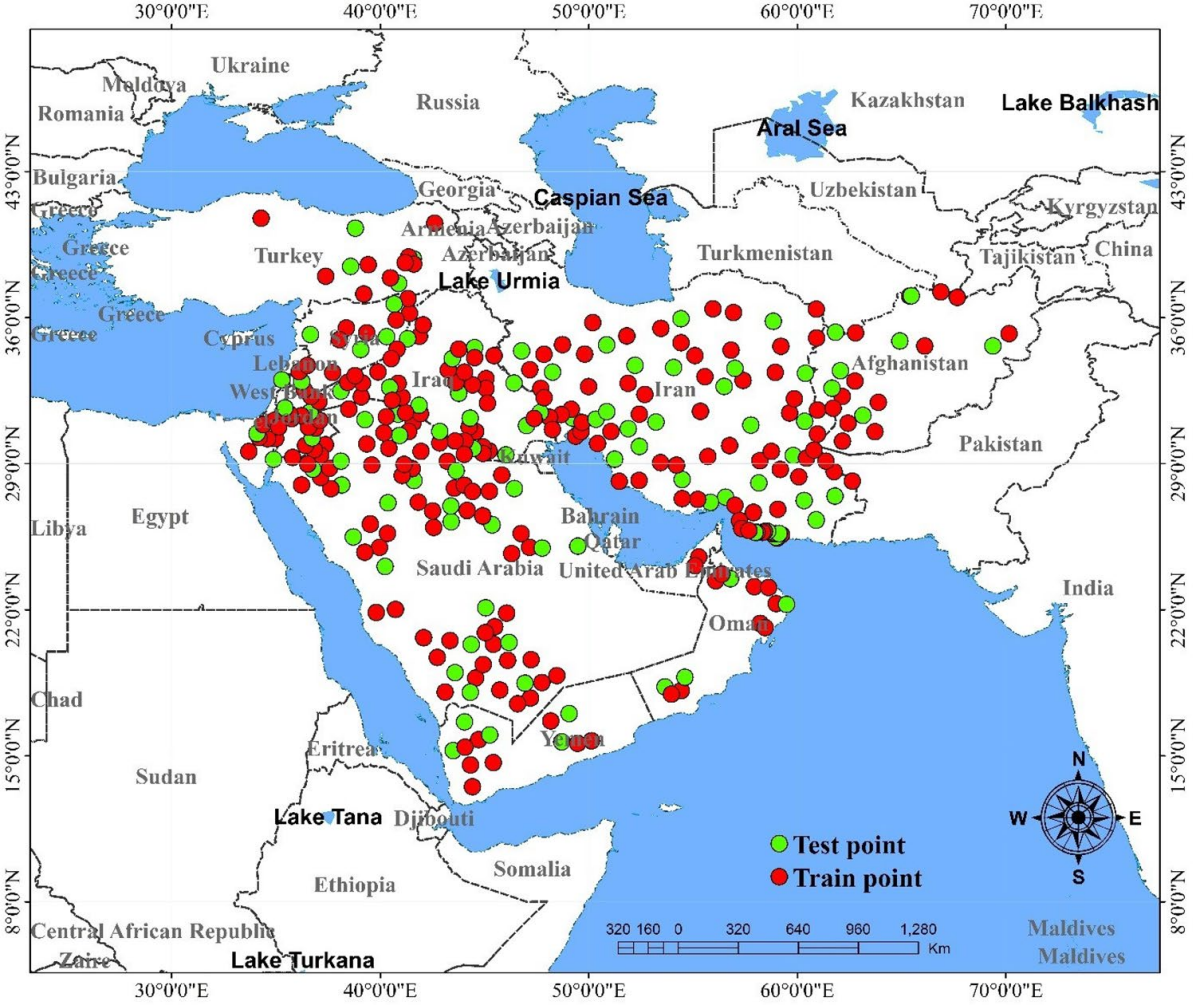
The location of the train and test samples in the study area—the ME. This map was generated in ArcGIS 10.4.1 (https://www.esri.com/en-us/about/about-esri/overview).

For building the predictive DL models of the dust sources, an inventory map^10^—a map showing current sources of dust—is needed. To obtain it, we consulted the inventory of dust sources provided by Boroughani et al.^15^ and the World Bank^27^. Predictive DL models were then constructed by dividing datasets randomly into train (70% or 222 samples) and test procedures (30% or 99 samples) (Fig. 2).

### Feature selection (FS) by the Dragonfly algorithm (DA)

FS is a main stage in the spatial modeling by data mining^28^. The goal of this step is to select the smallest subset of relevant variables (features) from a dataset that captures data characteristics for the generation of an adequate classification, which can be achieved by eliminating repeated, unnecessary, or noisy variables. This procedure speeds up data mining models and improves their performance.

DA—a swarm-based optimization technique—imitates the hunting and migration mechanisms of idealized dragonflies^29^. DA as a swarm intelligence, metaheuristic algorithm^30^ include five key steps and all five principles require control of five variables, including separation (*S*_*i*_), alignment (*A*_*i*_), cohesion (*C*_*i*_), attraction towards the food source (*F*_*i*_), and distraction from the enemy (*E*_*i*_).

1 $${S}_{i}=- \sum_{j=1}^{N}X- {X}_{i}$$

2 $${A}_{i}=\frac{{\sum }_{j=1}^{N}{V}_{j}}{N}$$

3 $${C}_{i}=\frac{{\sum }_{j=1}^{N}{x}_{j}}{N}-X$$

4 $${F}_{i}={F}_{loc}-X$$

5 $${E}_{i}={E}_{loc}+X$$

where *V*_*j*_*, F*_*loc*_*,* and *E*_*loc*_ indicate the speed of the jth neighbor, the position of the food source, and the enemy’s position, respectively; and *X*_*i*_ is the population (*i* = 1, 2, …, *n*). We used the ‘*zoofs’* library to run the DA method.

Successful applications to solve various optimization problems by DA have been reported by several authors^31–33^, but there are no reports to date on the application of this method for features controlling wind erosion and dust sources.

### DL hybrid models for prediction of dust sources

#### CNN-GRU hybrid model

CNNs—the best architectures of neural networks that learn a hierarchy of complex features by sequential convolution, pooling, and activation function^34^—were initially designed for image classification and recognition, but are today also used in different fields of computer science such as image super-resolution, semantic segmentation, localization, object detection, etc.^35,36^. Due to the high ability of CNNs to learn problem-specific features from raw input data, this architecture mainly reduces the need for handcrafted features^36^.

GRU—an updated version of LSTM—consists of update (*z*_*t*_) and reset (*r*_*t*_) gates^37–39^. GRU optimizes the LSTM network while maintaining LSTM performance. The update and reset gates are used to control the extent to which the information of the previous moment is brought into the current moment and to control the degree of ignoring the information of the previous moment, respectively. *r*_*t*_ and *z*_*t*_ determine how the new input is combined with the last memory and how much of the previous memory is to be retained, respectively. *r*_*t*_, *z*_*t*_ are new memories (*h*_*ti*_) and hidden states (*h*_*t*_) that can be expressed as follows:

6 $$ r_{t} = \, \sigma \, \left( {{\text{W}}^{{({\text{r}})}} {\text{x}}_{{\text{t}}} + {\text{ U}}^{{({\text{r}})}} {\text{h}}_{{{\text{t}} - {1}}} } \right) $$

7 $$ {\text{z}}_{{\text{t}}} = \, \sigma \, \left( {{\text{W}}^{{({\text{z}})}} {\text{x}}_{{\text{t}}} + {\text{ U}}^{{({\text{z}})}} {\text{h}}_{{{\text{t}} - {1}}} } \right) $$

8 $$ h_{ti} = {\text{ tanh }}\left( {{\text{r}}_{{\text{t}}} {\text{o Uh}}_{{{\text{t}} - {1}}} + {\text{ Wx}}_{{\text{t}}} } \right) $$

9 $$ h_{t} = \, \left( {{1 }{-}{\text{ z}}_{{\text{t}}} } \right){\text{ o h}}_{{{\text{ti}}}} + {\text{ z}}_{{\text{t}}} {\text{o h}}_{{{\text{t}} - {1}}} $$

where σ(…) is a sigmoidal function, and W^(r)^ and W^(z)^ are weight matrices, while o is the element-wise product.

**Figure 3:**
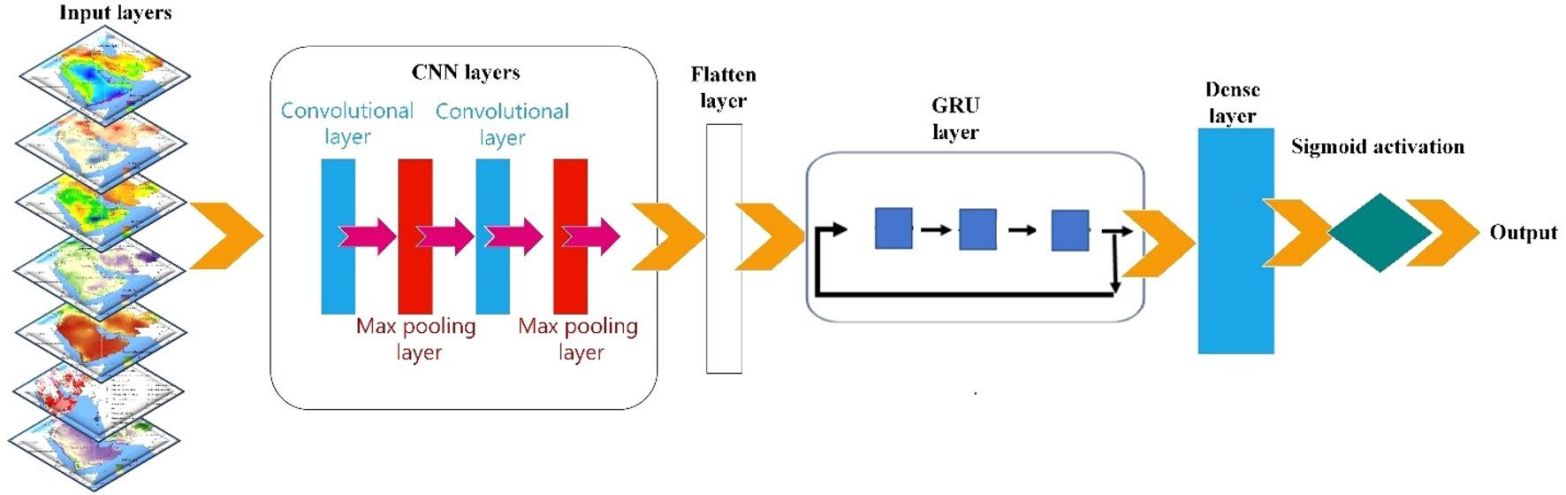
The structure of the CNN-GRU network for predicting dust source.

Here, we present our newly-designed CNN-GRU model (Fig. 3) for the classification of the dust sources in the ME and provide a detailed description of the hyper-parameters by which the model is tuned. Grid-search technique is used to find the optimal hyper-parameters of a model which results in the most ‘accurate’ predictions. At this method, the parameters of the estimator used to apply these methods are optimized by cross-validated grid-search over a parameter grid.

The network structure of the CNN-GRU model has nine layers: (1) one-dimensional convolution or convolution1D (with input dim = 7, number of neurons = 128, and with the rectifier linear unit (ReLU) as activation function), (2) max-pooling layer, (3) convolution1D (with neuron number = 64 and the activation function = ReLU), (4) max-pooling layer, (5) flatten layer, (6) GRU layer with the neuron number equal to 32, (7) dropout layer with a value of 0.25, (8) GRU layer (number of neurons = 16), and (9) fully connected layer with the neuron number = 1. At the compilation stage, the binary cross-entropy is selected as the loss function. The Adam optimization algorithm^40^ was chosen as optimizer because the model’s accuracy with this optimizer was higher than that with Adamax. The metrics function denotes the accuracy (metrics function = accuracy). At the fitting stage of the model, among different numbers of the epoch (200, 500, and 1000) and batch size (10, 15, and 20), the model with epoch = 1000, and batch size = 10 performed better than other possible combinations. The ReLU activation function (g(z)) and its derivative (g(z)ʼ) can be expressed as:

10 $$ {\text{g}}\left( {\text{z}} \right) \, = {\text{ max }}\left( {0,{\text{ z}}} \right) $$

11 $$ {\text{g}}\left( {\text{z}} \right)^{\prime } = = \left\{ {\begin{array}{*{20}c} {0, z < 0 } \\ {1, z > 1} \\ \end{array} } \right. $$

#### Dense layer deep learning (DLDL)—random forest (RF) (DLDL-RF) hybrid model

**Figure 4:**
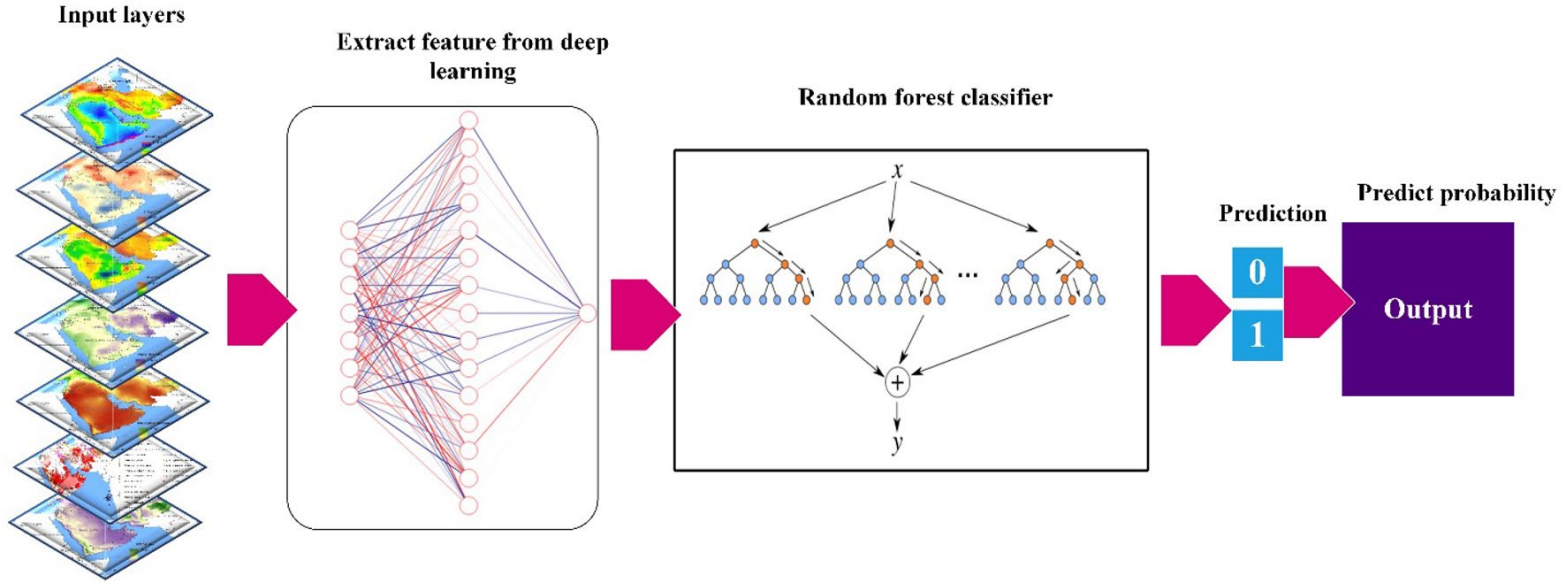
Conceptual diagram for the DLDL-RF model for prediction of dust sources.

We introduce a new DL hybrid model (DLDL-RF) based on the dense layer deep learning (DLDL) or fully connected layer and random forest (RF)^41^ models (Fig. 4) to classify dust sources in the ME.

New data were generated by DLDL, which were then entered into the RF model to classify the dust sources. Here, we describe the hyper-parameter tuning of the dense-RF model. The structure of the model’s network includes three dense layers. In the first dense layer, the input dim is equal to seven, the number of neurons is 30 and ReLU is the activation function. In the second dense layer, the number of neurons is 15 and ReLU is selected as an activation function. The third dense layer has a neuron number of 1 and a sigmoidal activation function. At the compilation stage of the dense-RF model, the loss function, optimizer and metrics function are binary cross-entropy, Adam, and accuracy functions, respectively. At the model’s fitting stage, the number of the epoch and batch size were selected as 3000 and 10, respectively, because the model with this epoch number and batch size performed better than other combinations. Finally, all parameters for both models were adjusted by the grid-search automat method.

### Assessment of the performance and interpretability of DL hybrid models

The model’s predictive performance was assessed by six statistical criteria consisting of accuracy, precision, recall, F1 score, Cohens kappa, and receiver operating characteristic—area under curve (ROC-AUC). The game theory through the permutation feature importance measure (PFIM) suggested by Breiman (2001) determines the importance of the influential variables for controlling dust source. A variable is “important” if permutation of its values increases the model error relative to the other features, and a feature is “unimportant” if permutation of its values keeps the model error relatively unchanged^14^.

**Figure 5:**
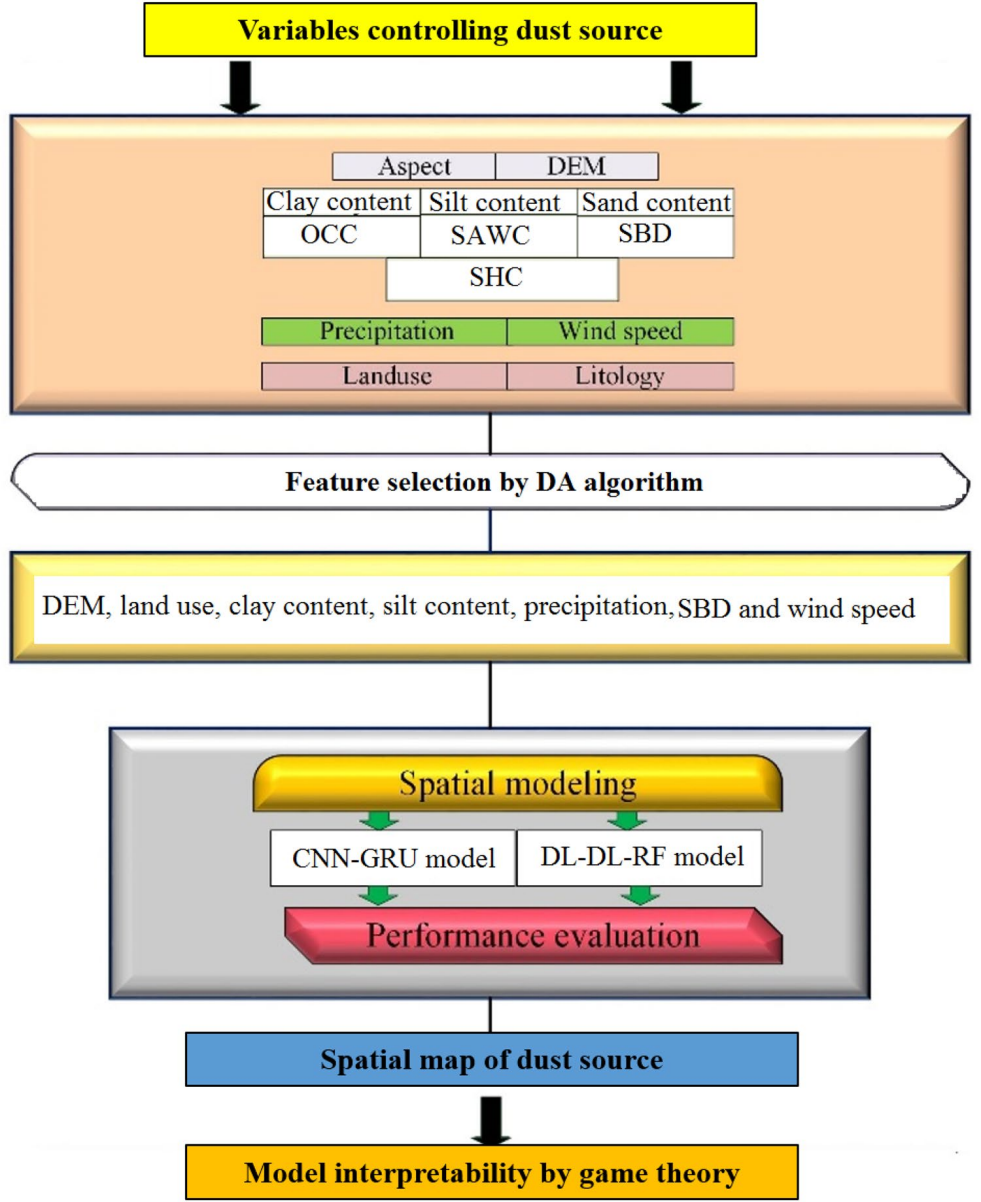
Flowchart of the main stages for mapping dust sources by DL modeling and game theory. OCC, SAWC, SBD and SHC indicate the organic carbon content, soil available water content, soil bulk density, and saturated hydraulic conductivity, respectively.

All of the key steps for the spatial modeling of dust sources by feature selection, DL models, and game theory are presented in Fig. 5.

## Results and discussion

### Important variables controlling dust source

Many environmental, geographic, and bioclimatic variables consist of soil properties, digital elevation models (DEM), topographic characteristics, vegetation cover, land surface roughness, wind conditions, precipitation, and slope which together control dust sources or dust emissions from the land surfaces^4,10,13,26^. Therefore, discriminating important features from the non-important ones by feature selection is a key step for a successful modeling process that can serve to increase the accuracy of predictive models.

**Figure 6:**
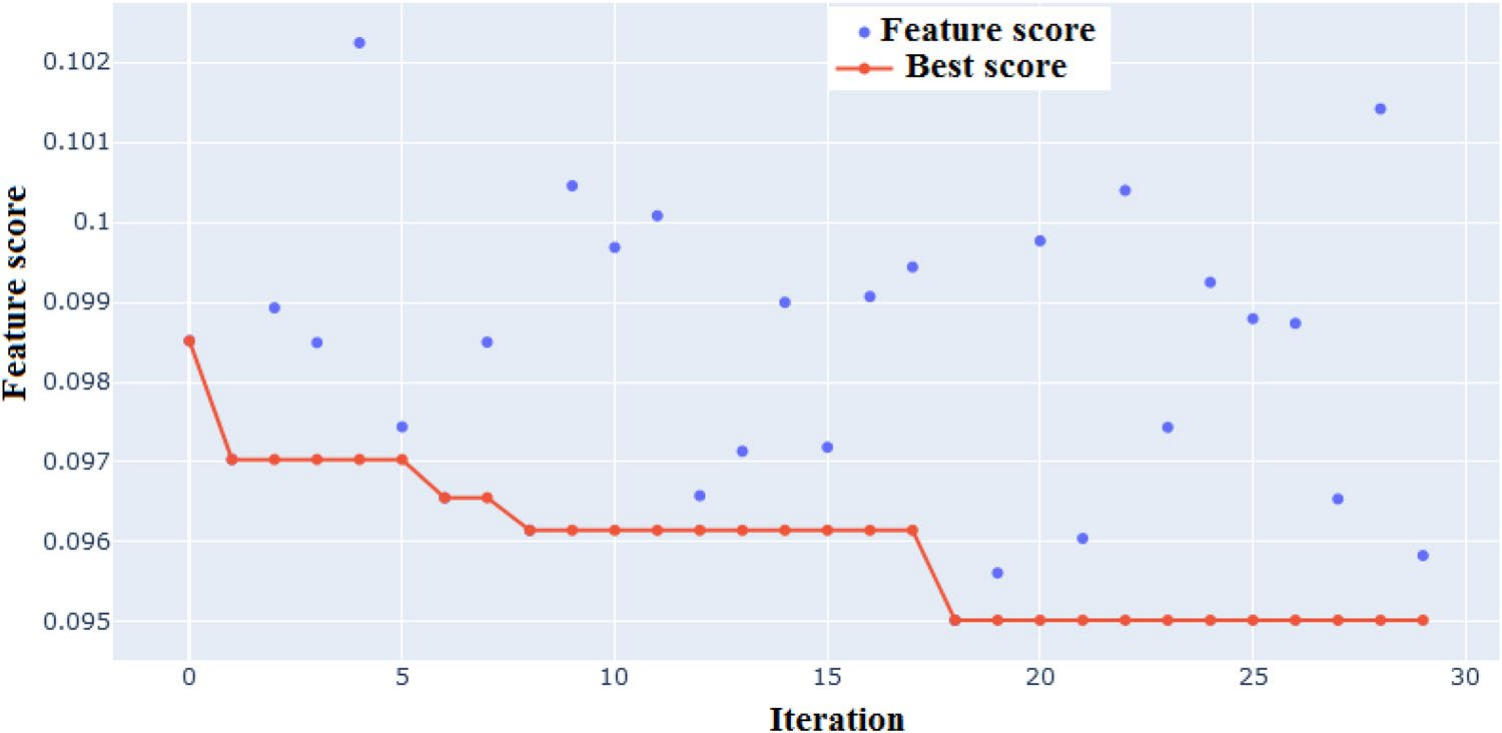
Feature selection by DA.

Features selected by DA are presented in Fig. 6. Among the 13 features explained as potential variables controlling dust sources, seven features consisting of DEM or elevation, land use, clay content, silt content, precipitation, soil bulk density and wind speed were selected as important variables by DA, the model having the lowest error when these features were included in the model. The objective function is optimized when the difference between observed and predicted values is the lowest, i.e. where the lowest value of the objective function is observed when the seven above mentioned variables are included in the model. According to Fig. 6, the lowest value of the objective function occurred after 18 iterations. Precipitation, soil bulk density and slope were selected as the three most important variables in Central Asia^10^. Three critical variables controlling dust source in the Sistan basin comprise wind speed, elevation and soil organic carbon, all three having been identified by a genetic algorithm^41^.

### Spatial maps of dust source

**Figure 7:**
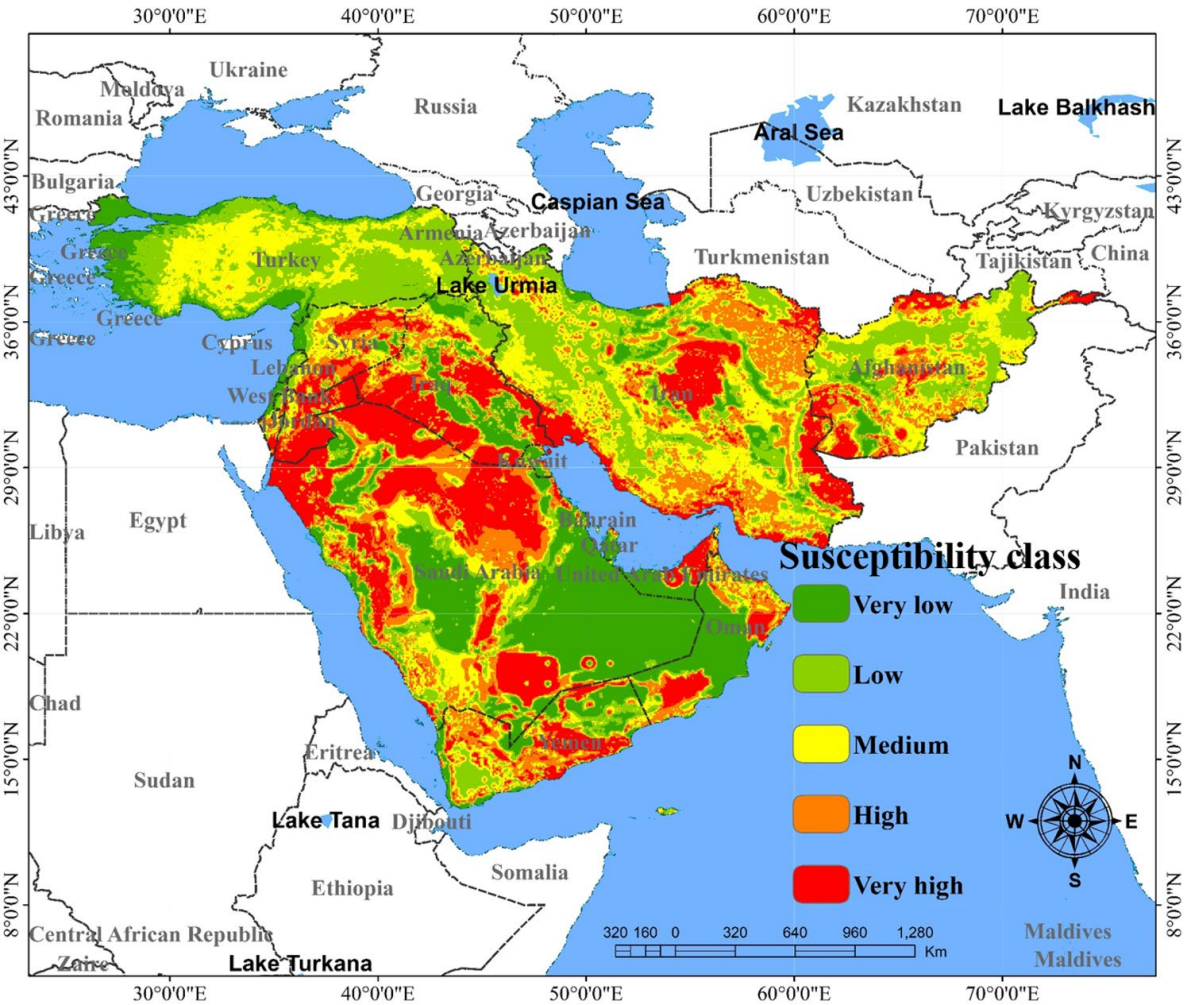
The spatial map of dust sources generated by the CNN-GRU hybrid model. The values for pixels was predicted in python, and then, values of pixels were mapped by ArcGIS 10.4.1 (https://www.esri.com/en-us/about/about-esri/overview).

**Figure 8:**
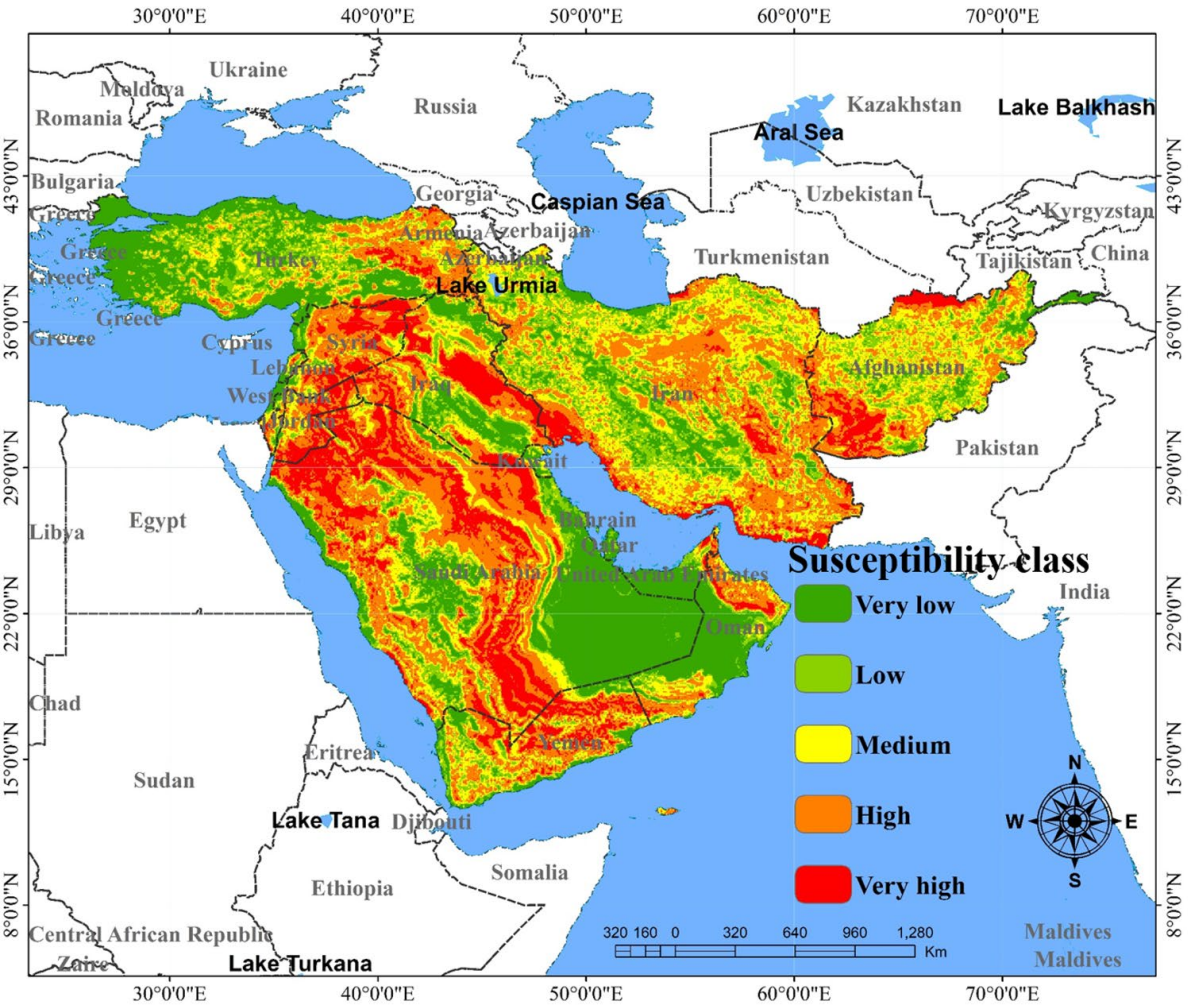
The spatial map of dust sources generated by the DLDL-RF hybrid model. The values for pixels was predicted in python, and then, values of pixels were mapped by ArcGIS 10.4.1 (https://www.esri.com/en-us/about/about-esri/overview).

The spatial maps of dust sources produced by CNN-GRU and DLDL-RF are presented in Figs. 7 and 8. The area of land susceptibility classes as dust sources predicted by the two DL hybrids are shown in Table 1. The predictions related to the land susceptibility classes as dust sources can be divided into four classes^10^, or five classes. In the present study the land susceptibility was divided into five classes (very low, low, moderate, high and very high). Based on the results, the spatial map of dust sources generated by the CNN-GRU hybrid model suggested that 19.7, 21.7, and 22.9% of the study area were classified as having very low, low and moderate susceptibility, whereas 17.2 and 18.9% of the area were classified as having high and very high susceptibility, respectively (Fig. 7). The results of the DLDL-RF model predicted that 23.1, 22.8, 22.2, 20.2, and 11.7% of the total area belonged to the low, moderate, high and very high susceptibility classes, respectively (Fig. 8). Based on the two models, more than 31% of the total area is classified as having high and very high susceptibility. Overall, the most important dust sources in the study area can be divided into 12 regions consisting of southeastern Iran and the border of Iran/Afghanistan (Sistan plain), central Iran (e.g., Yazd, Esfahan and Semnan provinces), southwestern Iran (especially the southern parts of Khuzestan province), a minor source in northern Qatar, a minor source in the middle of Kuwait, eastern Iraq and the border of Iran/Iraq (especially the desiccated bed of the Hawizeh Marshes), border of Iraq/Saudi Arabia (southern Iraq and northern Saudi Arabia), northern Syria, eastern Jordan (or border of Jordan/Saudi Arabia), central Yemen, northern United Arab Emirates, and southwestern Oman.

**Table 1:** The area of dust source susceptibility classes predicted by the CNN-GRU and DLDL-RF models.

Predictive DL hybrid model	Area	Susceptibility class				
		Very low	Low	Moderate	High	Very high
CNN-GRU	Km^2^	1,318,370	1,453,116	1,533,600	1,150,028	1,237,576
	%	19.7	21.7	22.9	17.2	18.5
DLDL-RF	Km^2^	1,545,082	1,527,326	1,484,457	1,350,140	785,685
	%	23.1	22.8	22.2	20.2	11.7

A large area of the ME consisting of arid and semi-arid climates, is impacted by dust storms^25^. This region—ME—has several dust sources such as Saudi Arabia, Syria, Iraq, and Iran^42^. According to the analysis of TOMS data by^26^, the ME is one of the key sources for generating dust particles on earth. Based on the satellite remote sensing and visibility data taken from meteorological stations, Khuzestan province and the Sistan basin are the most dust-affected regions in Iran^5,43^, delivering consistent results in the present study. Recent droughts in external dust storm source areas (Iraq and Syria sources) have increased dust events in western Iran^44^.

Arabia is identified as one of the five key world regions where dust storms frequently occur^45^. A dust source with more significant extension and lesser intensity is located in northeastern Saudi Arabia to the north of the great sand sea—Rub Al Khali^26^–, which is consistent with our results. The land along the eastern shore of the Red Sea or the western part of Saudi Arabia can be another primary source in this country. Dust storms frequently occur in the capital of this country—Riyadh–, where the visibility decreases markedly over many days^26^. Based on the meteorological data analyzed by^46,47^, the highest frequency of dust events was observed in southern Iraq and Kuwait. The most intense dust storms in Qatar, Kuwait, and Iraq occur from April to August each year^26^.

### Assessment of the DL hybrid model’s performance

Table 2 presents the results of the predictive DL hybrid models performance assessment for both datasets (train and test) by six statistical indicators. Based on all six indicators used to assess the model’s performance, the accuracy of the DLDL-RF hybrid model performed slightly better for both datasets than did the CNN-GRU model. A hybrid approach by synthesizing ≥ two models into an overall model can improve the prediction accuracy and provide reasonably good results^48^. The deep learning architecture with a low false-positive diagnostic rate performed better than machine learning and shallow neural networks^49^. For example, Gholami et al.^10^ reported that the copula-gcForest hybrid model (as a DL model) performed better than the individual DL models (e.g., gcForest and Bi-LSTM) for mapping different classes of dust sources in Central Asia. DL models (RNN and RBM) as novel techniques in aeolian geomorphology generate accurate spatial maps of dust sources to help target mitigation of detrimental dust effects on climate, ecosystems and human health^16^.

**Table 2:** The values of the six statistical indices for assessing the DL model’s performance based on train and test datasets.

Criteria	Train dataset		Test dataset	
	CNN-GRU	DLDL-RF	CNN-GRU	DLDL-RF
Accuracy	90.8	99.8	87	96
Precision	92.7	100	90	97
Recall	79.7	99.5	84	94
F1 score	85.7	99.8	87	95.4
Cohens kappa	79	99.6	74	92.4
ROC-AUC	88	99.8	87	96.2

Until today, applications of CNN-GRU and DLDL-RF hybrid models for spatial modeling of dust sources have not been reported, but there are a limited number of studies that have applied the CNN to the temporal prediction of dust and PM_10_. For example, more recently, Sharma et al.^50^ reported that their DL hybrid model or CNN-GRU model outperformed other models for forecasting PM_10_. A new DL hybrid model—CSVR—based on CNN and support-vector-regression (SVR) proposed by Chimire et al.^51^ with lower root mean square error (RMSE) and mean absolute error (MAE) performed better than DL and machine learning models (e.g., LSTM, DBN, MARS, RBF, etc.) for solar radiation prediction. Zhang et al.^52^ introduced a CNN-GRU model based on multi-task deep learning for the prediction of air quality. They reported that their proposed model had good temporal stability and generalization ability. In comparison with different DL models (e.g., deep belief network (DBN), deep neural network, artificial neural network, etc.), a DL hybrid model consisting of CNN, an extreme gradient boosting with RF, and a Harris Hawks Optimization, was more efficient for predicting boosting solar radiation^53^. The spatial map of soil salinity generated by a one-dimensional convolution neural network—long short-term memory (1DCNN-LSTM) DL hybrid model was more accurate than the salinity map produced by deep Boltzmann machine (DBM) DL individual model^54^.

### Interpretability of DL models by game theory

**Figure 9:**
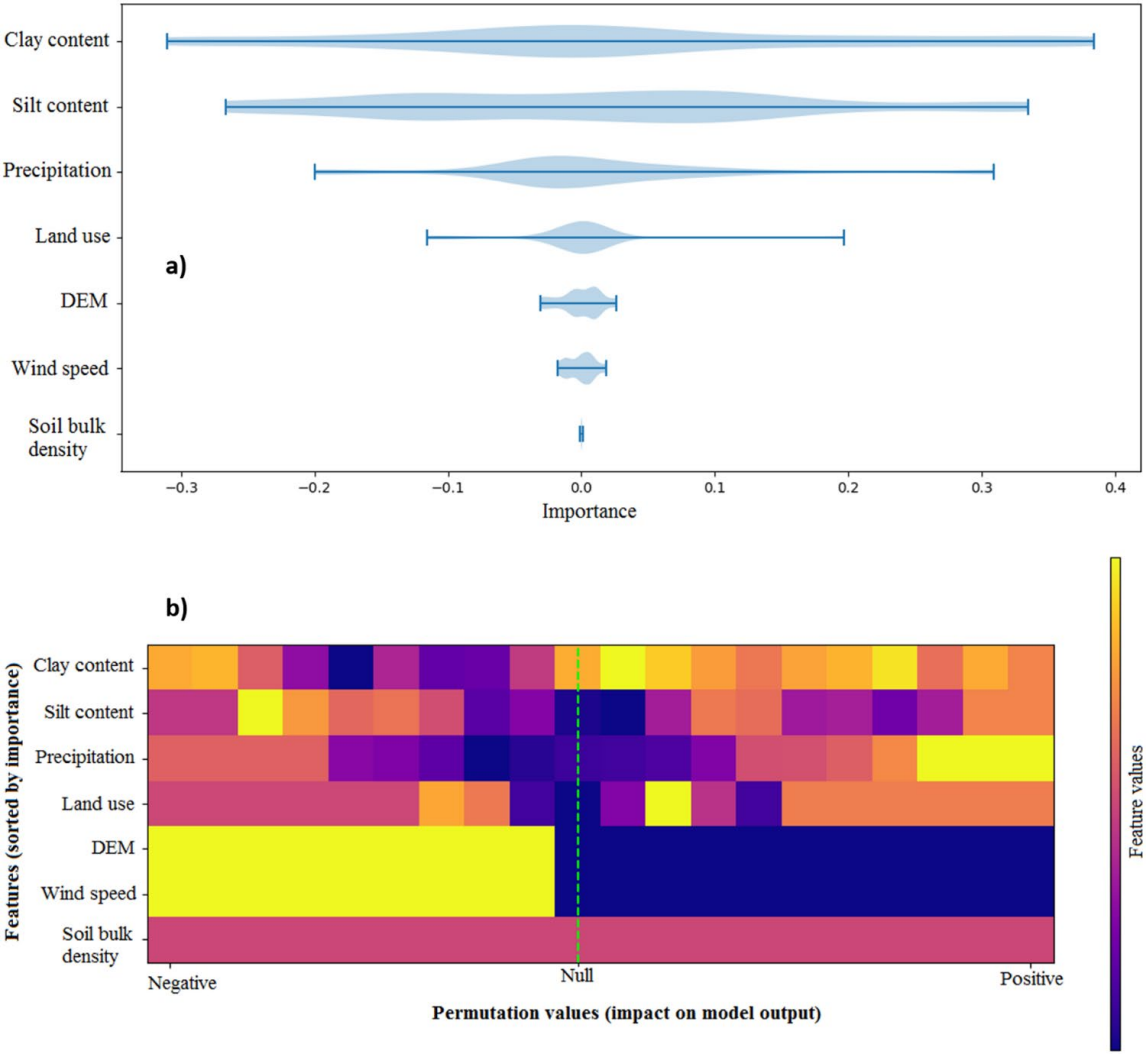
The importance and permutation values for the important features (variables) controlling dust sources calculated by game theory.

The relative importance of the important variables controlling dust sources and their contribution determined by game theory are presented in Fig. 9. Among seven important features selected by DA, three features (e.g., clay content, silt content, and precipitation) have the highest importance scores and, based on the permutation values, have the highest contribution or impact on model output. Overall, pedo-climatic variables, land surface conditions and roughness are the important variables controlling wind erosion and dust emissions^55,56^.

## Conclusion

This study is the first attempt to develop two DL hybrid models (CNN-GRU and DLDL-RF) for the classification of dust sources in the ME. DA and game theory were applied to the feature selection and interpretability of the DL hybrid models, respectively. Both DL hybrid models performed well for the classification of dust sources, but the DLDL-RF hybrid model performed slightly better than the CNN-GRU hybrid model.

In comparison with existing techniques (e.g., sediment source fingerprinting, geochemical source tracing methods, field-based methods and physical models) for studying dust sources, our DL hybrid models are valuable tools for the classification of dust sources (very low, low, moderate, high, and very high) at large spatial scales (e.g., country, continent, and globe). They can also be applied to other regions experiencing frequent dust storms such as southwestern Asia, northern Africa, the arid and semi-arid regions of USA, Australia, and areas with poor ground-based observation datasets, particularly as the models are inexpensive and do not require any field sampling and laboratory measurements. The main limitations for research are related to the features used in the modeling process. For example, additional accuracy for models may be achieved with higher spatial resolution of the imagery, and more detailed soil maps. Overall, we recommend that future research apply CNNs with various sequential learning mechanisms to the spatial mapping of different environmental hazards.

## Supplementary Information

Supplementary Information.

## Data Availability

The datasets used and/or analysed during the current study available from the corresponding author on reasonable request.
