# Supplementary material for: Novel deep learning hybrid models (CNN-GRU and DLDL-RF) for the susceptibility classification of dust sources in the Middle East: a global source
Source: Sci Rep. 2022 Nov 11;12:19342. doi: 10.1038/s41598-022-24036-5 (PMC9652306; doi:10.1038/s41598-022-24036-5)
Supplement: Supplementary file 1 — Supplementary Information. [file 41598_2022_24036_MOESM1_ESM.docx]

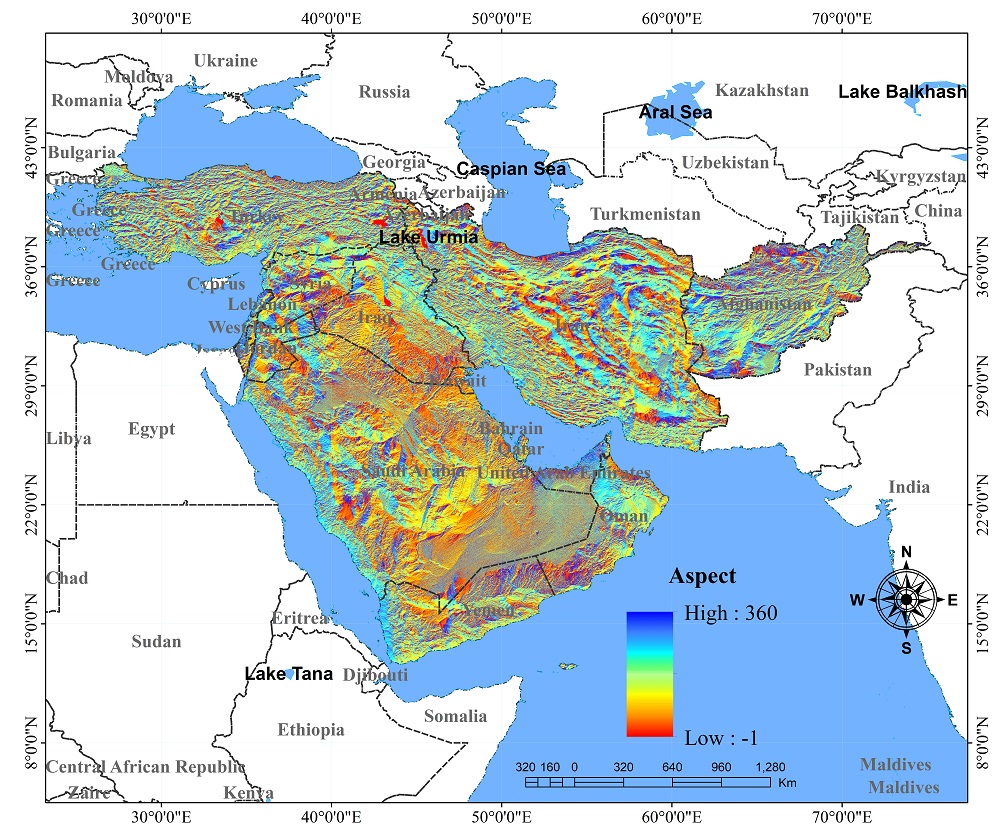


S1. Aspect map for the study area.


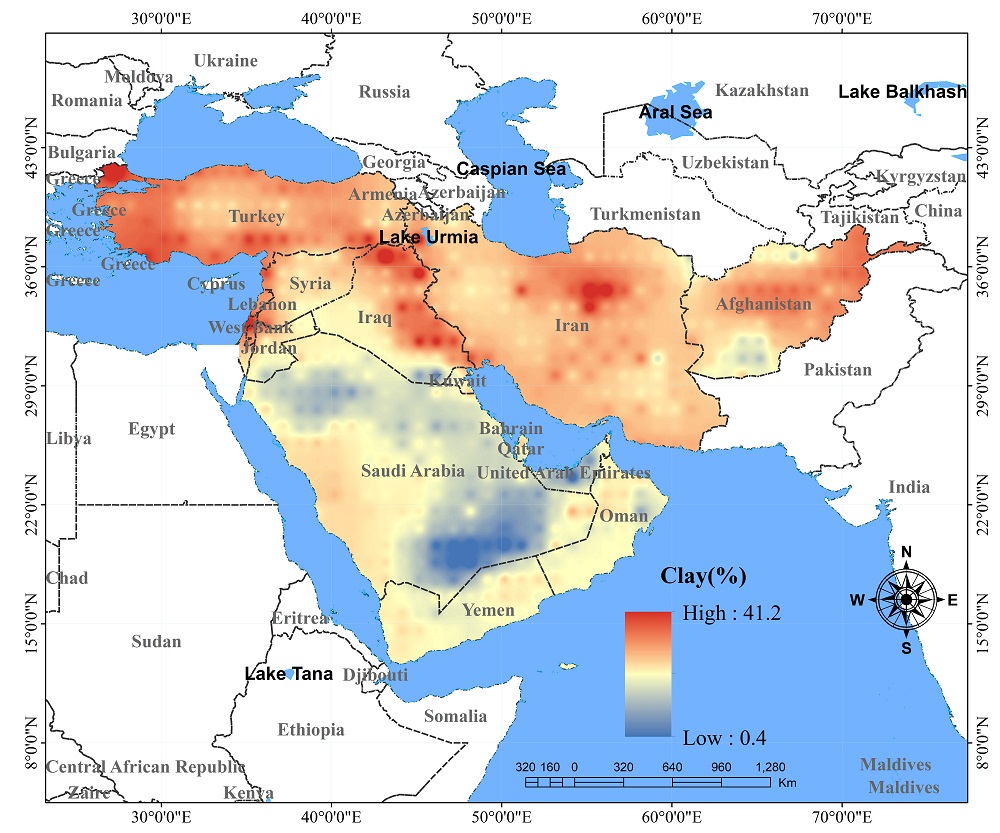


S2: Clay content in the study area.


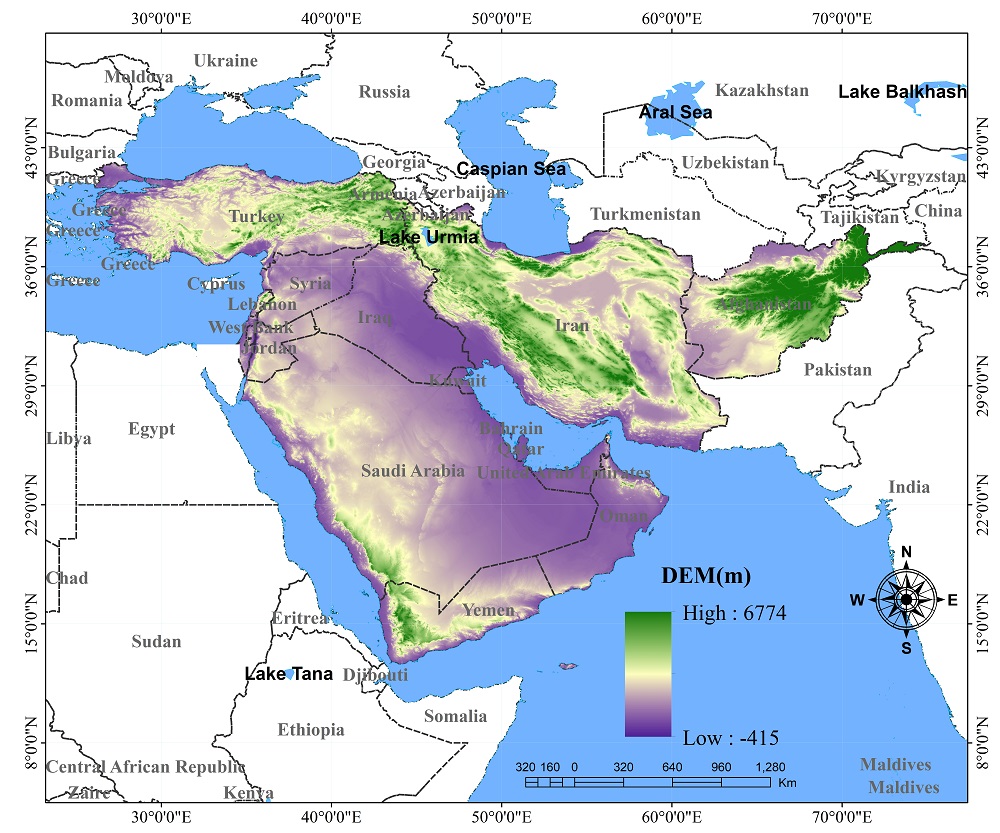


S3: DEM for the study area.


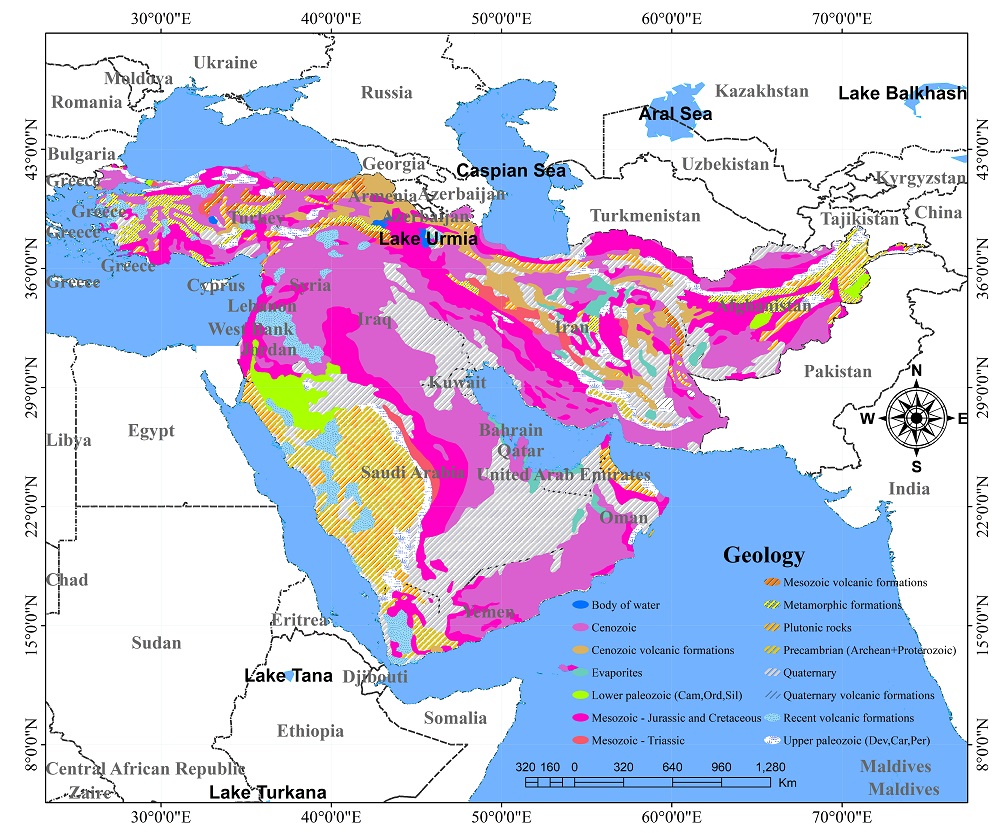


S4: Lithology map of study area.


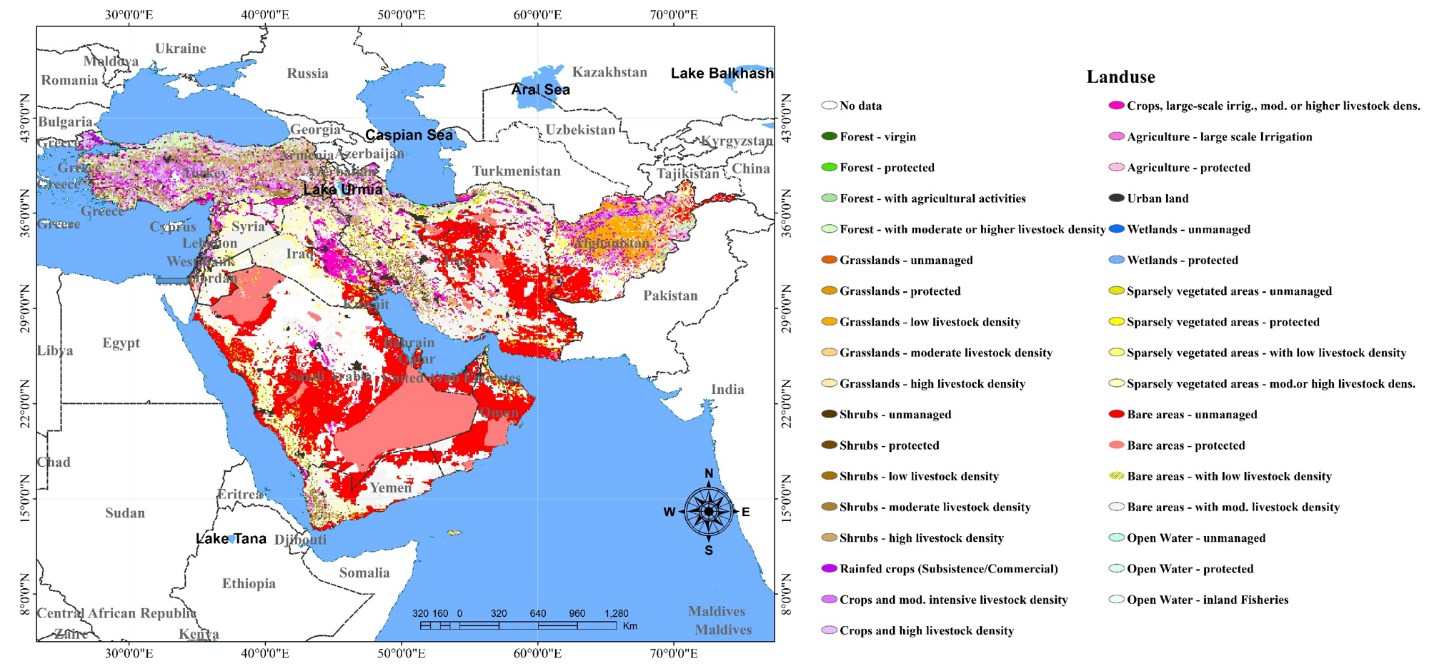


S5: Land use map for the study area.


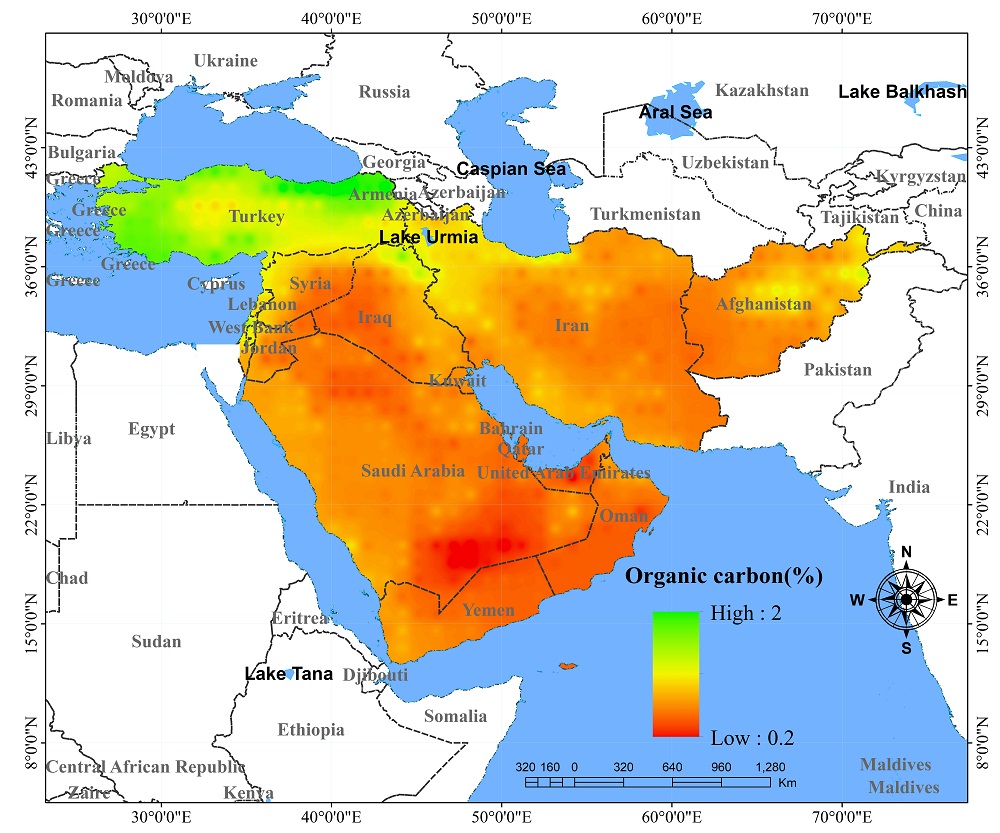


S6: organic carbon content in the study area.


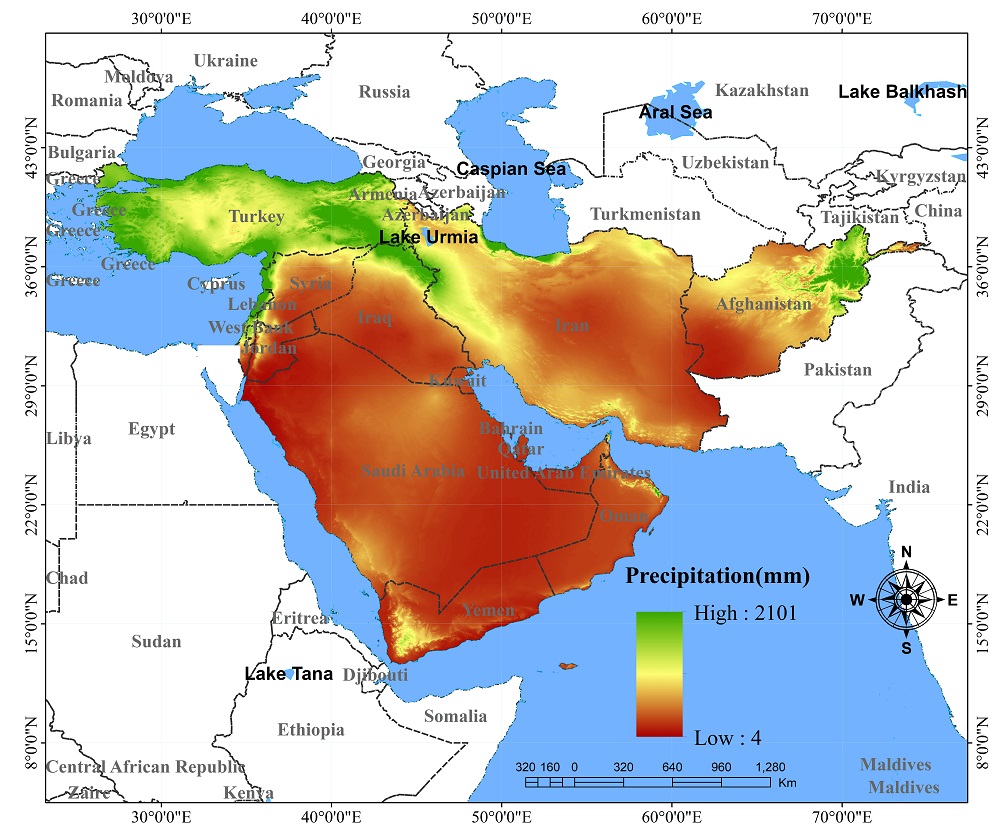


S7: Precipitation map of study area.


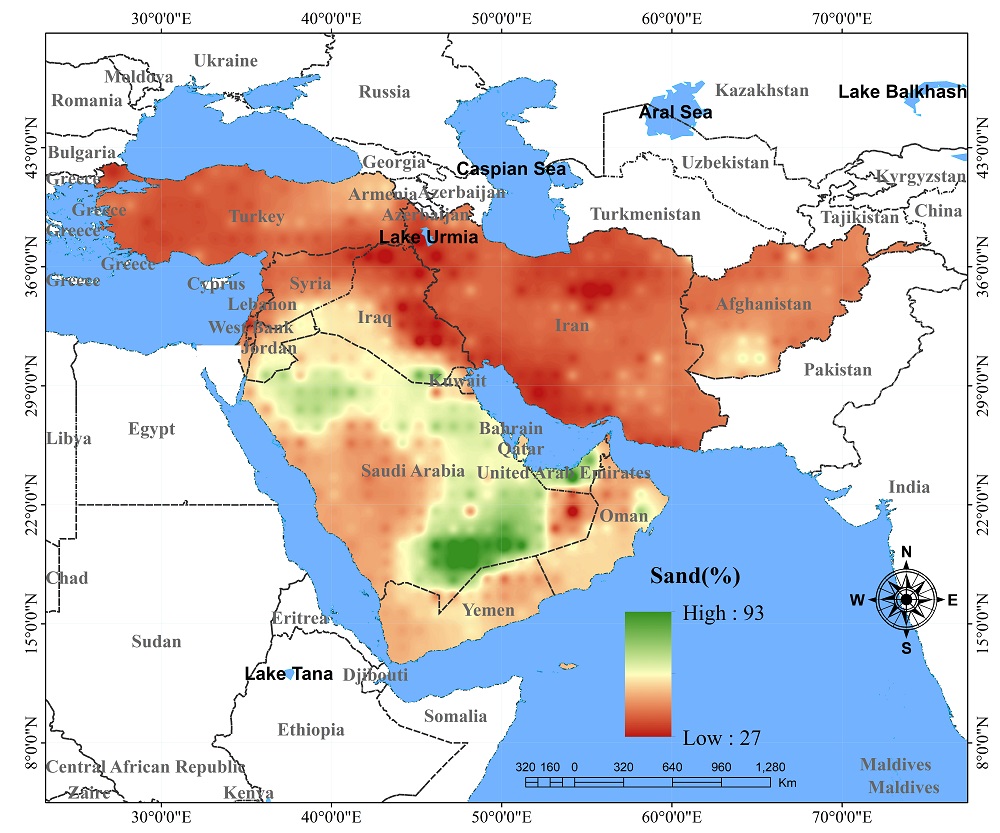


S8: Sand content in the study area.


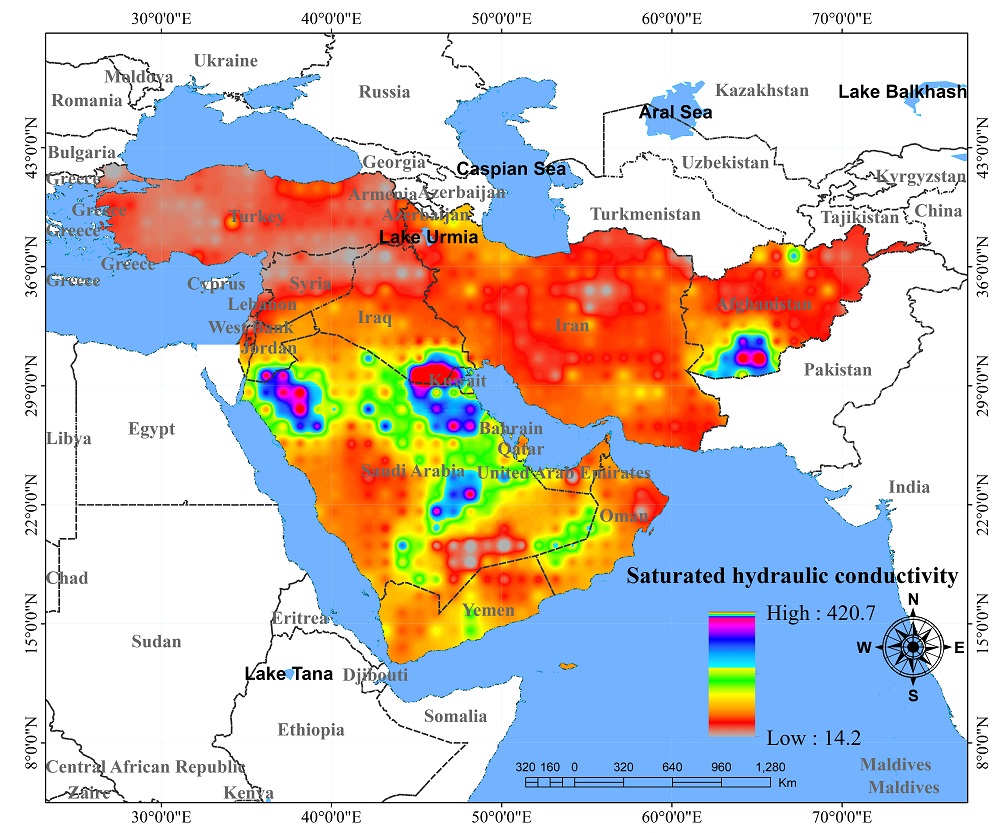


S9: Saturated hydraulic conductivity of soil in the study area.


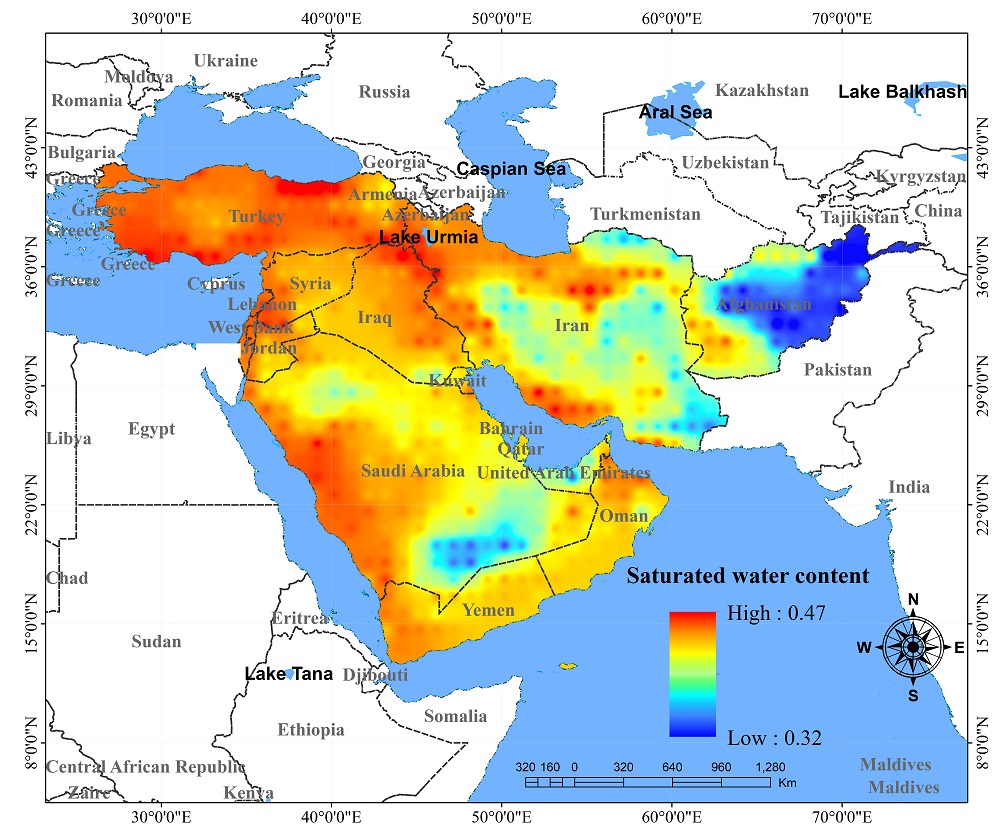


S10: Saturated water content of soil in the study area.


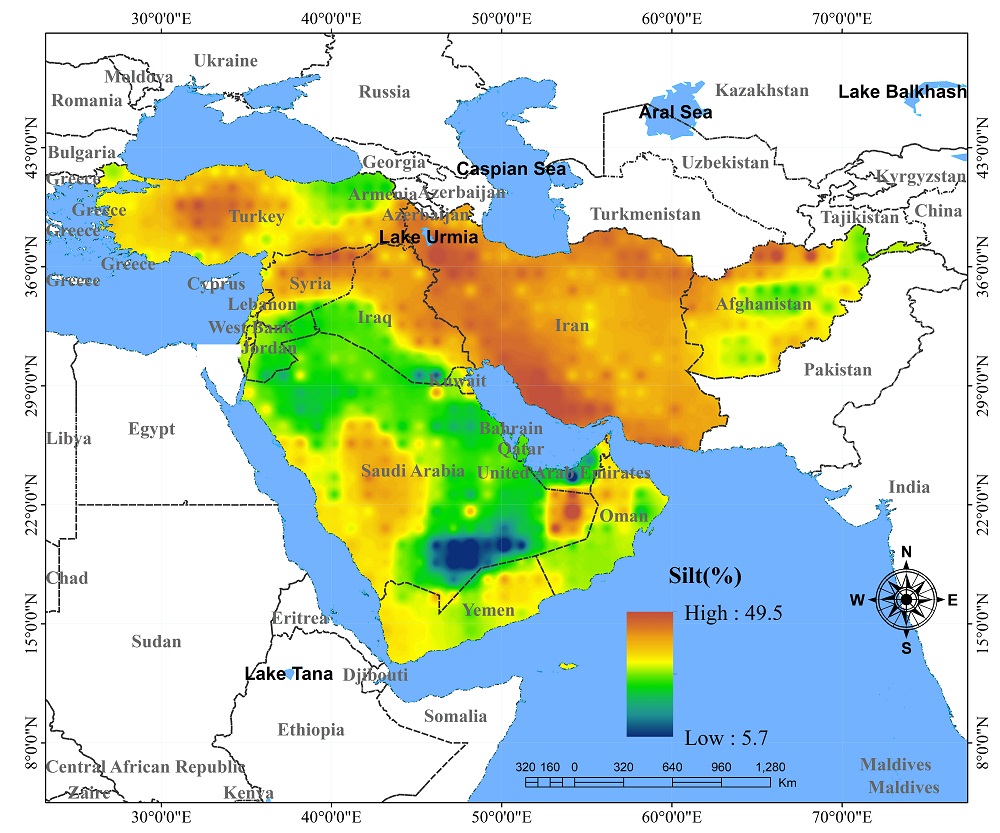


S11: Silt content in the study area.


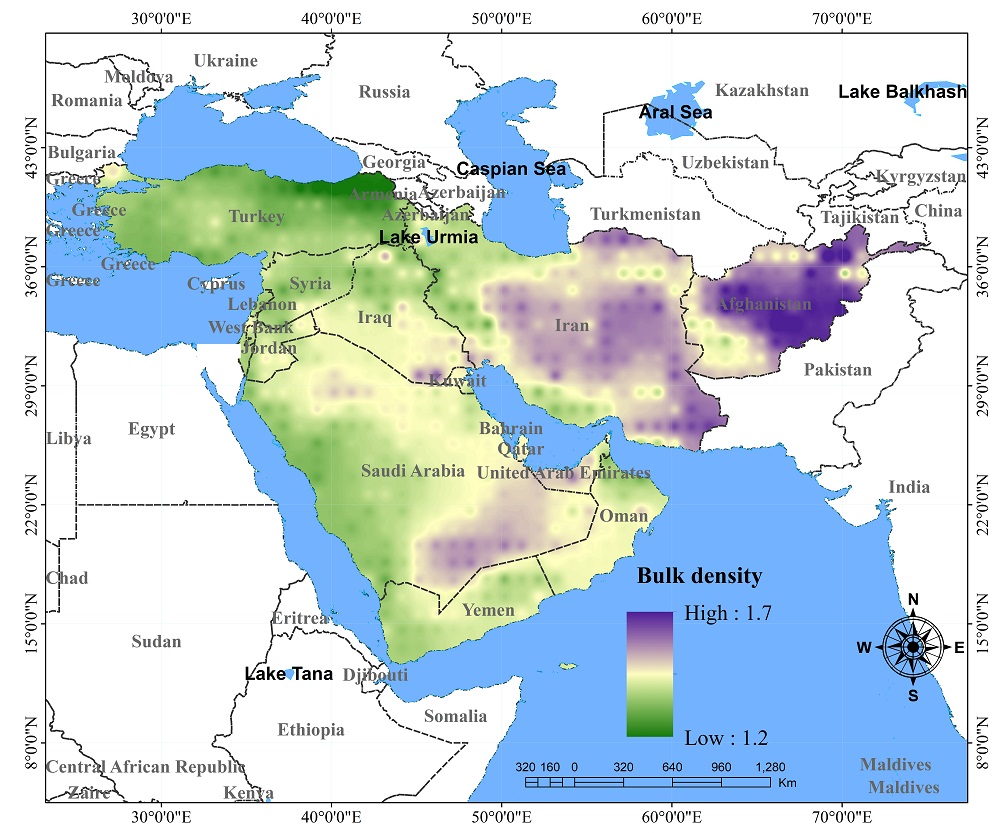


S12: Soil bulk density in the study area.


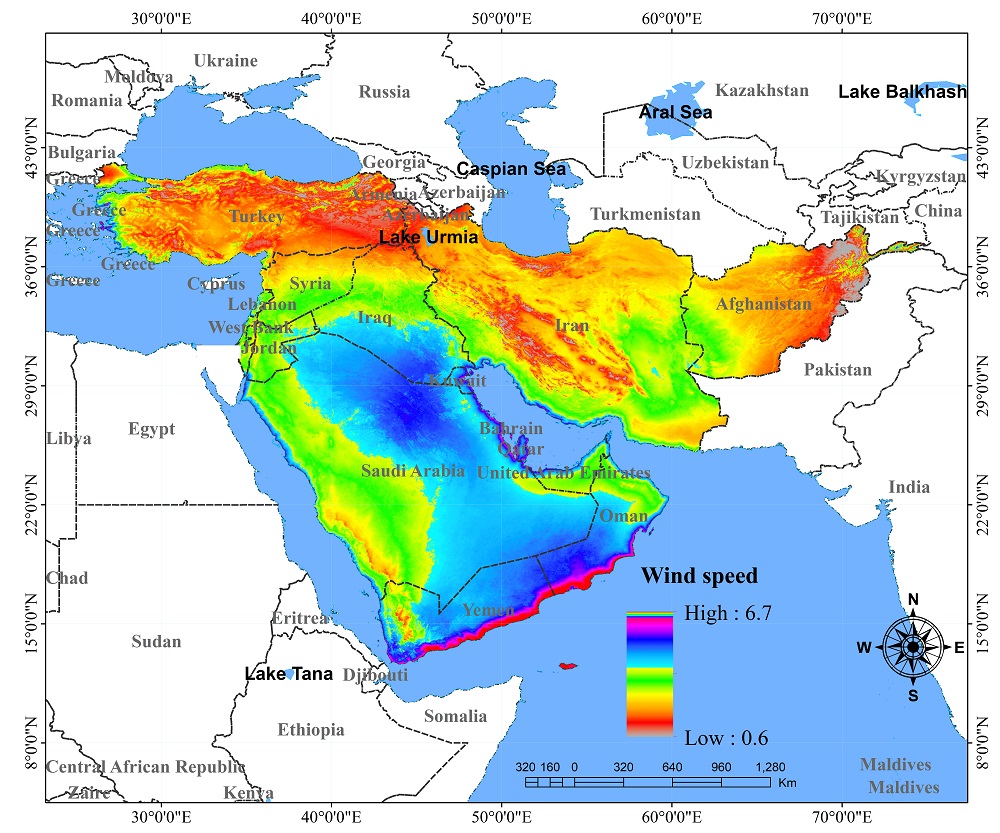


S13: Wind speed map of study area.
